# Chemical Profiling of Wild and Commercial Tomato Leaves: Protocol Optimization, Insights into Differences in Chemical Profiles between Species and Developmental Stages

**DOI:** 10.1021/acsomega.5c03637

**Published:** 2025-09-18

**Authors:** Maria Clara Santana Aguiar, Moacir Rossi Forim

**Affiliations:** Department of Chemistry, 67828 Universidade Federal de São Carlos, Rod. Washington Luiz Km 235 s/n, 13565-905 São Carlos, São Paulo, Brazil

## Abstract

The tomato is a globally important crop whose domestication has led to significant chemical changes that may reduce its natural defenses. This study compares the chemical profiles of wild (*Solanum pimpinellifolium*) and commercial (*Solanum lycopersicum*, cultivar BRS Iracema) tomato leaves across three developmental stages: seedling, vegetative, and flowering. We used optimized ultrasound-assisted extraction for nonvolatile compounds and headspace extraction for volatile compounds. We coupled these methods with UHPLC-q-TOF-MS/MS and GC–MS to investigate their chemical composition. Multivariate analysis revealed that domestication profoundly altered chemical profiles, particularly affecting glycosylated alkaloids, phenylpropanoids, and gibberellins. During the seedling stage, *S. pimpinellifolium* exhibited higher accumulation of defense-related compounds, including alpha-tomatine and tomatidine. In contrast, the commercial species exhibited enhanced anthocyanin biosynthesis and higher levels of glyceric acid, particularly during the vegetative stage. Differences in specific compounds, such as 3-indoleacrylic acid, certain peptides, clausarinol, and gibberellin aldehyde A12, were also observed across developmental stages. While chemical profiles varied across developmental stages, the impact of domestication on the tomato chemical profile was generally more pronounced. These findings provide information for targeted breeding, such as the identification of key metabolites like alpha-tomatine and tomatidine that enhance stress resistance. The findings also help to identify the biosynthesis of anthocyanins and other compounds that improve the nutritional quality of cultivated tomato species.

## Graphical abstract

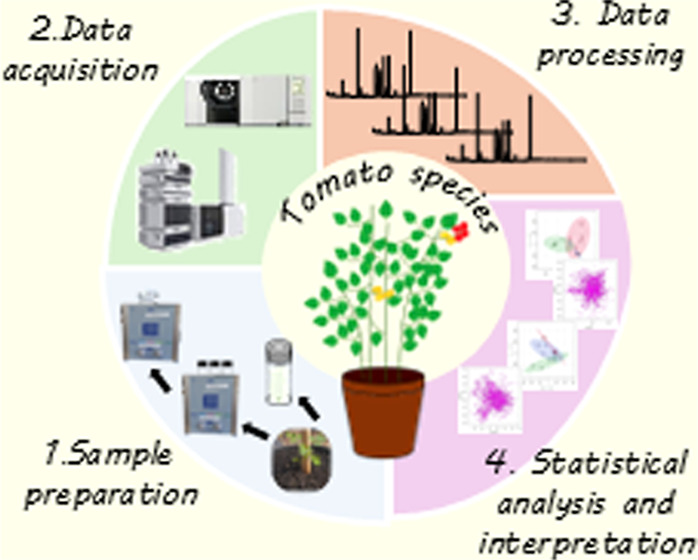

## Introduction

The tomato is one of the world’s most important agricultural crops. It is widely valued for its nutrients and bioactive compounds, including carotenoids and antioxidants.^1–3^ Originally from the Andes, the tomato underwent an intense domestication process over generations. This resulted in the development of varieties with desirable agronomic traits, such as larger fruits and higher yields.^4–7^

However, this artificial selection focused on specific traits often reduces the genetic diversity of cultivated plants.^4,5^ This decrease in genetic variability compromises the natural defenses of plants, making modern crops more susceptible to diseases, pests, and the impacts of climate change.^8,9^ Salazar-Mendoza et al. (2023)^10^ presented a practical example. In this study, the researchers demonstrated that the cultivated tomato species *Solanum lycopersicum* showed greater susceptibility to the specialist herbivore *Tuta absoluta* than its wild relatives did. Furthermore, domestication has been associated with the loss of important secondary compounds, affecting the flavor and aroma profiles of commercial varieties compared to their wild ancestors.^7,8^

However, when a range of tomato genotypes—from wild relatives to modern cultivars—were exposed to various pests and diseases in a more complex context, Ferrero et al. (2020)^11^ concluded that differences in performance and the effects of these pests and diseases were not directly related to the degree of domestication or genetic kinship. Additionally, Jaiswal et al. (2020)^12^ demonstrated that domestication impacts not only the tomato’s direct defenses but also its interaction with beneficial soil microorganisms, which are crucial for systemic resistance. These findings suggest that the interactions among plants, pests, and environments are much more complex, and that defense mechanisms have diverse origins, evolving throughout natural and artificial selection processes involving both plant genetics and ecological interactions.

Therefore, domestication acts within this already complex system, inducing chemical changes in plants that are not fully understood.^12^ This knowledge gap is particularly evident in the variation of these chemical defenses throughout the plant’s developmental stages because domestication may compromise the plant’s ability to respond naturally to different biotic pressures at various points in its life.^13,14^

In this context, studies such as those by Reimer et al. (2021)^15^ and Alfosea-Simón et al. (2022),^16^ have investigated leaf metabolism in tomato species under stress conditions and in different phenological phases. However, a more comprehensive and detailed comparative analysis is needed. Characterizing the chemical profiles of wild and commercial species, including volatile and nonvolatile compounds, across multiple developmental stages is an indispensable step in deepening our understanding of the impact of domestication on plants.

The primary objective of this study was to investigate the chemical changes induced by domestication in tomato plants, address the existing gaps among species, and to create a comprehensive chemical profile. Specifically, the study aimed to identify differences between the wild species *Solanum pimpinellifolium* and the commercial species *S. lycopersicum*, cultivar BRS Iracema. To this end, two complementary experiments were conducted. The first experiment examined on nonvolatile compounds using ultrasound-assisted solid–liquid extraction (UAE). The second experiment focused on volatile compounds and employed static headspace extraction (HS). UAE efficiently recovers nonvolatile compounds and offers advantages such as speed and high efficiency compared to conventional extraction methods.^17,18^ HS is an automated technique that minimizes sample preparation and ensures that only volatile molecules are analyzed by the chromatographic system. This significantly reduces matrix effects and optimizes the analysis of volatile compounds.^19,20^

Both methods were optimized and the extracts were analyzed using ultrahigh-performance liquid chromatography (UHPLC) and gas chromatography coupled with mass spectrometry (GC–MS) to comprehensively analyze the chemical profiles. The integrated approach of these experiments provided a more complete understanding of how domestication altered the chemical composition of tomatoes at different developmental stages, from seedlings to flowering.^21^ These findings offer valuable insights for developing genetic breeding strategies that reintroduce lost defensive and quality characteristics into commercial tomato species.

## Materials and Methods

### Reagents

We prepared a caffeine stock solution at 100 μg·mL^–1^ (Sigma-Aldrich, St. Louis, USA) with water and kept it at 8 °C. We also prepared a bisabolol solution at 500 μg·mL^–1^ (Sigma-Aldrich) with glycerol. Caffeine and bisabolol were used as internal standards in liquid and gas chromatography, respectively. Solvents used included methanol, HPLC-grade dichloromethane (Supelco, North Carolina, USA), pyridine (99.8% v·v^–1^, Sigma-Aldrich), glycerol (Synth, São Paulo, Brazil), and ultrapure water (Milli-Q, Millipore, Merck KGaA, Darmstadt, Germany).

LC–MS grade formic acid (Fluka, Missouri, USA) was used in the mobile phase of the liquid chromatography system. For sample preparation, *N*,*O*-bis­(trimethylsilyl)­trifluoroacetamide (BSTFA) with 1% chlorotrimethylsilane (TMCS) (Sigma-Aldrich) was used for primary metabolites. Sodium chloride from Synth, São Paulo, Brazil, was used for headspace extraction.

### Plant Material

We obtained *S. pimpinellifolium* seeds (PI access 126925) from the Embrapa Hortaliças germplasm bank in Brasília, Brazil. We obtained the high-yielding, pathogen-tolerant cherry tomato hybrid BRS Iracema (*S. lycopersicum*) from local stores.^22^ This study was registered in SisGen (Sistema Nacional de Gestão do Patrimônio Genético e do Conhecimento Tradicional Associado). The registration number is AFF721B.

We have grown the plants in a greenhouse with transparent netting. This protected them from pests and external threats. The plants received natural light from May to July 2023. They experienced about 12 h of light and 12 h of darkness. The average temperature was 30 °C and the humidity was about 40 ± 20%. Germination of the seeds was started in trays. After 30 days they were transferred to 4.5 L pots. The pots contained a mixture of vegetable soil, sand, and earthworm humus in a 3:1:1 ratio. To prevent water stress, the plants were watered daily to maintain consistent soil moisture levels of 60%. Soil moisture was monitored with a digital soil moisture meter, PHD-3000 (SVE Super). We added 1 m bamboo stakes to the pots to support the plants.

### Collection of Tomato Leaf Samples

We investigated how different growth stages affect metabolite production. Leaf samples were collected at three key phenological points: (i) seedling: samples were collected when the first two true leaves fully appeared, about 10 days after sowing. (ii) Vegetative: Samples were collected when the plants had two complete lateral stems, approximately 30 days after sowing. Each stem had seven leaves. At this stage, we picked the youngest leaves from the first stem. We chose the leaves that were closest to the top of the stem. (iii) Flowering: Samples were collected samples after the first flower emerged, around 50 days after sowing. We collected the youngest leaves from the stem closest to the apex.

Three leaves were harvested from each of five individual plants at each sampling stage. Although the described phenological markers (e.g., appearance of true leaves, lateral stems, and first flower) were consistent indicators for sampling across both species, slight differences in timing (in days after sowing) to reach these stages may have occurred between *S. pimpinellifolium* and *S. lycopersicum* (cultivar BRS Iracema) due to their distinct growth rates and developmental patterns. However, sampling was strictly guided by the defined morphological criteria for each stage.

The samples were quickly frozen in liquid nitrogen to stop their metabolism. They were then shipped to the laboratory and divided into two batches. The first batch was lyophilized using Liotop L101 (Liobras, São Paulo, Brazil) and then powdered with an 80-mesh sieve. This batch, intended for extracting nonvolatile metabolites, was stored at −20 °C. The second batch was stored in a −80 °C Sanyo MDF-U56VC ultrafreezer (Panasonic, Osaka, Japan) for volatile metabolite extraction.

### Metabolite Extraction Protocols

To ensure robust and reproducible chemical profiling, two distinct extraction protocols were optimized using a composite sample containing equal amounts of leaves from all species and developmental stages. We evaluated the efficiency of each extraction method based on two primary criteria: the total number of compounds detected and the sum of their peak areas in the respective chromatographic analyses. The independent variables selected for optimization were based on established protocols from our research group,^23^ Kwon et al. (2019),^24^ and Mun et al. (2021).^25^

### Optimization of Nonvolatile Metabolite Extraction Using Ultrasound-Assisted Extraction (UAE)

Ultrasound-assisted solid–liquid extraction (UAE) was optimized to recover nonvolatile metabolites from dried tomato leaves. A 2^2^ factorial design with four center points was used to study the impact of two independent variables: extraction time (5, 20, and 35 min) and ethanol concentration in water (30%, 50%, and 70% v·v^–1^). The planning matrix can be seen in the Table S1. For each extraction experiment, 2.5 mg of lyophilized and powdered plant material was weighed into an 8 mL glass tube. Then, 500 μL of the designated ethanol solution was added (as per the factorial design). Extraction was performed using an Elma ultrasonic bath (Tovatech, Germany), operating at 60 kHz.

The extraction procedure involved two consecutive cycles. After the first cycle, the supernatant was carefully collected. Then, an additional 500 μL of the same extraction mixture was added to the remaining precipitate for a second extraction cycle under the same conditions. The two cycles’ resulting supernatants were then combined. The pooled extract was then centrifuged at 3200 *×g* for 30 min at 10 °C to remove particulate matter. Next, it was filtered through a 0.200 μm PVDF membrane. For UHPLC-q-TOF-MS/MS analysis, a 10 μL aliquot of the filtered extract was diluted with ultrapure water to a final volume of 500 μL. Finally, 10 μL of a 100 μg·L^–1^ caffeine solution (internal standard) was added to each diluted sample.

### Derivatization for Primary Metabolite Analysis by GC–MS

To analyze primary (nonvolatile) metabolites by GC–MS, transfer 50 μL of the combined UAE extract (before the final dilution for UHPLC-q-TOF-MS/MS) to a 0.300 mL micro reaction flask. The solvent was completely evaporated under a gentle stream of nitrogen. Then, 60 μL of pyridine was added, followed by 100 μL of the derivatization reagent *N*,*O*-bis­(trimethylsilyl)­trifluoroacetamide (BSTFA) with 1% chlorotrimethylsilane (TMCS). The mixture was heated at 60 °C for 30 min in a G4023D Gehaka drying oven (São Paulo, Brazil). After derivatization, the extracts were diluted with 40 μL of dichloromethane and filtered through a 0.22 μm PTFE membrane prior to GC–MS analysis using a Shimadzu GC–MS TQ-8040.

### Optimization of Volatile Metabolite Extraction (Static Headspace—HS)

Static headspace (HS) extraction was optimized for isolating volatile organic compounds from fresh tomato leaves. This method was optimized using a 2^2^ factorial design with four center points. Two independent variables were investigated: extraction time (5, 15, and 25 min) and ionic strength. Ionic strength was adjusted by adding 500 μL of NaCl solution at three concentrations: 0%, 15%, and 30% (m·v^–1^). The obtained matrix for planning can be seen in the Table S3. For each extraction, 100 mg of frozen fresh plant material was placed in a 10 mL headspace vial. The material was kept frozen at −80 °C until the time of sample preparation. Then, a 25 μL aliquot of a 500 mg·L^–1^ bisabolol working solution (prepared in glycerol) was added as an internal standard. The vials were securely sealed with an aluminum crimp cap fitted with a PTFE/SIL septum. The analysis was performed using a 2.5 mL PALSyr HS for Combi-PAL system. Headspace extraction was performed at 75 °C with an agitation speed of 500 rpm. Then, 1000 μL of the vapor phase was injected into a Shimadzu GC–MS TQ-8040.

### Chromatographic Analyses

We analyzed nonvolatile organic compounds using an ultrahigh performance liquid chromatography system (Agilent 1290). We used a Zorbax Eclipse Plus phenyl-hexyl column as the stationary phase (2.1 × 100 mm and had a particle size of 1.8 μm). The column oven was maintained at 40 °C and the autosampler at 10 °C. We used a constant flow rate of 0.300 mL·min^–1^ with gradient elution. Solvent A was 0.1% formic acid in water and solvent B was 0.1% formic acid in methanol. The gradient elution program was as follows: 15% B increased linearly to 85% B from 0 to 9 min, held at 90% B from 9 to 14 min, and returned to 15% B from 14 to 15 min. A 5 min postrun was included for column re-equilibration. The injection volume was 5.0 μL. A quadrupole time-of-flight mass spectrometer (Agilent 6545 Q-TOF MS) was used for ionization and analysis. It had an electrospray ionization (ESI) source operating in positive ion mode. The capillary voltage was set at 3.5 kV. The source temperature was 320 °C. The desolvation gas was set at 350 °C. It flowed at 11 L·min^–1^. Cone gas flow was 8 L·min^–1^. The fragmentor voltage was 250 V. The skimmer voltage was 65 V, and the nozzle voltage was 0 V. We acquired MS data in the mass range of 50 to 1700 Da.

The derivatized compounds were analyzed using a Shimadzu gas chromatograph (GC-2010 Plus) and a TQ-8040 triple quadrupole mass spectrometer. A DB-5 IU capillary column (Agilent) was used for this analysis (stationary phase of 5% diphenyl and 95% dimethylpolysiloxane, 30 m × 0.25 mm inner diameter and a layer thickness of 0.25 μm). Helium 5.0 was used as the carrier gas at a flow rate of 1.10 mL·min^–1^. The chromatographic conditions were as follows: injector temperature at 280 °C; initial column temperature at 100 °C held for 4 min. The temperature was then increased at a rate of 10 °C·min^–1^ to 290 °C and held at this temperature for 11 min. The transfer line temperature was 280 °C and the ion source was at 200 °C. We injected 1.0 μL in split mode (10:1). We recorded electron impact mass spectra using an ionization energy of 70 eV. The mass range of the acquisition quadrupole was 45 to 600 Da. Data were acquired in scan mode at a rate of 0.3 scans·s^–1^.

Volatile organic compounds were also analyzed using a Shimadzu gas chromatograph (GC-2010 Plus). A VF-Wax MS capillary column (Agilent) was used for this analysis (polyethylene glycol stationary phase, 30 m × 0.25 mm inner diameter and a layer thickness of 0.25 μm). Helium 5.0 was used as the carrier gas at a flow rate of 1.50 mL·min^–1^. The chromatographic parameters were: Injector temperature 180 °C. The initial column temperature was 40 °C for 1 min. It was then ramped up at 10 °C per minute to 230 °C and held for 4 min. The interface temperature was 230 °C. The ion source was at 270 °C. 1000 μL was injected (splitless mode). The quadrupole mass analyzer operated in electron ionization mode at 70 eV. It collected data in the mass range of 45 to 500 Da at a rate of 0.3 scans per second. We injected quality control samples from all varieties. We did this with the internal standard solution. This helped to check the stability of the analytical systems.

### Data Processing Methods

We processed mass spectrometry data from liquid chromatography using the Molecular Feature Extraction algorithm (Mass Hunter Qualitative Analysis Workflows B.08.00 software from Agilent Technologies). This process involved the alignment of retention times and the definition of molecular features. These features included retention time, mass (with isotopes, charge states, adducts, and neutral losses), and quantitative data such as peak area or intensity. The resulting molecular feature data were tabulated. Molecular feature annotation involved comparing our data to entries in databases such as METLIN, MassBank, and *m*/*z* Cloud. We also reviewed relevant studies published in the scientific literature. For analysis of data obtained from the gas chromatography system, the raw data were converted to CSV format. We identified compounds by comparing the spectral data to the NIST library (version 17.0).

After collecting key information from each platform, we combined the data from different treatments and formed a matrix. This improved our predictions and made the results easier to understand.^26,27^ The matrix was sized and then used for further statistical calculations in Mass Profiler Professional 15.0 software from Agilent Technologies. In this data processing step, the high-resolution data were recalibrated based on the exact mass of the internal standard, with a mass tolerance of 2 ppm. A retention time alignment was also performed with a tolerance of 0.15 min. We centered the data by subtracting the mean value from each point. After processing the data, we converted the matrix to.CSV format. This allows visualization and analysis in the MetaboAnalyst platform (version 6.0).^28^

### Statistical Analysis

We used analysis of variance (ANOVA) to evaluate the area and number of compounds from the extraction optimization methods. We used *F*-test at 5% significance level and performed regression analysis. The purpose of these analyses was to determine if there were significant differences between the variables studied under different extraction conditions. For the analyses, we used the calculation routines of Pereira and Pereira-Filho (2018)^29^ together with the software Octave 4.4 (2018).

## Results and Discussion

### Development of Extraction Protocols

In this study, we examined two extraction methods: ultrasonic-assisted solid–liquid extraction (UAE) and static headspace extraction. Below, we describe the results of developing these extraction methods.

### Nonvolatile Organic Compounds in Tomato Leaves

We evaluated the extraction efficiency based on the number of extracted compounds and the total sum of peak areas. These data are presented in Table S1. Table S1 shows a 1.3-fold variation in molecular features. The lowest number was 2632, while the highest reached 3346. This difference occurred in both the first and second extraction cycles. The total area varied more than three times when looking at both extraction cycles. The lowest value was 2.99 × 109, while the highest was 9.24 × 109. The effect of each independent variable was based on the number of compounds from the first extraction cycle and the total peak areas from both cycles. Compounds from the second cycle were not counted. We did not find any unique molecular features in this cycle. However, more extractions result in better recovery of chemical compounds in the matrix.^30^ Therefore, we evaluated the total peak areas of the extracted compounds from both cycles.

The responses were converted to a dimensionless weight called individual desirability (d). To find the global desirability (D), we averaged the d values from each experiment.^31^ Table S1 shows these data. Desirability values range from 0 to 1. Values close to 1 are more desirable.^31^ Experiments at the center showed good desirability, scoring above 0.80. Experiments 1 and 4 had desirability scores above 0.44. This suggests the need for minor adjustments. Experiments 2 and 3 had values below 0.26, indicating low extraction efficiency. Based on these results, we constructed effect plots using the D data, as shown in Figure S1.

Figure S1 shows that the solvent mixture had a strong influence on the extraction process. In contrast, extraction time had little effect, accounting for less than 10%. Figure S1 shows that the extraction solvent mixture had a negative effect. Higher concentrations of organic solvents resulted in lower extraction yields. Similar results were also reported by Li et al. (2019)^17^ and Gao et al. (2021)^18^ using ethanol fractions around 60% (v·v^–1^) for polyphenol extraction. These results show that the polarity of the solvent affects how well we can extract metabolites. The polarity of the solvent must match the polarity of the target molecules.^18^ Our data show that reducing the ethanol concentration to 30% (v·v^–1^) decreases extraction efficiency. We found that extraction efficiency levels off at about 50% (v·v^–1^) ethanol in the solvent. Extraction time had little effect, but the best efficiency came from a 20 min extraction (see Table S1).

The results are clearer when looking at the chromatograms from Experiments 1 and 5–8. Figure S1 shows the molecular features from the first 5 min of elution. After deconvolution, the peak areas of the extracted metabolites increased. This was observed with a 60% (v·v^–1^) ethanol solvent and a 20 min extraction time.

We used ANOVA to select the best model for the experimental data. The results are presented in Table S2. The *r* ^2^ value was 0.9604. This means that the regression model explained over 96% of the response variability. A closer look showed that a regression model was not good for predicting values. The calculated *F*-value was found to be lower than the *F*-tab. The *F*-value should be about 10 times larger than the *F*-tab.^32^ We found a clear lack of fit. The results indicate that we should modify the scored intervals in the factorial design. The addition of axial points is also important.

Based on the data presented, we selected a 50% (v·v^–1^) ethanol solution and a 20 min extraction time in an ultrasonic bath for the extraction of fixed (nonvolatile) metabolites. We also performed two consecutive extraction cycles. We then evaporated the solvent and performed derivatization and analysis by GC–MS.

Figure S2 shows the chromatograms confirming the effectiveness of the extraction method for primary metabolite analysis. Derivatized samples showed fatty acids, organic acids, and carbohydrates, which facilitated species comparison. These methods effectively characterized the dynamic changes in the tomato metabolome during development.

### Volatile Organic Compounds in Tomato Leaves

Static headspace extraction is an automated method for isolating volatile compounds. It requires minimal sample preparation^19^ and ensures that only volatile molecules enter the injector of the chromatograph.^20^ Thus, it reduces steps and matrix effects compared to older methods. Given these advantages, this technique was evaluated in the present study. We investigated how ionic strength and extraction time affect the extraction of volatile molecules. We observed the number of compounds and peak areas shown in Table S3.

For the extraction of volatile organic compounds, the number of compounds varied from 9 to 29. The total peak area also changed from 9.02 × 10^5^ to 1.09 × 10^7^. This shows a 3-fold difference in the number of compounds and a 12-fold difference in the peak area. We converted these results into global desirability scores (Table S3). We then used them to calculate the effects of each variable (Figure S3).

Compared to the UAE results, headspace extraction at the central point showed low global desirability values of less than 0.20 and a relative standard deviation (RSD) of 21%. This suggests that the addition of NaCl solution improves both extraction efficiency and sensitivity. When an inorganic salt is added to a solution, it increases the ionic strength. As a result, organic compounds can be released more rapidly into the headspace.^33^ Headspace techniques have some limitations (e.g., septum quality and temperature control). Therefore, a RSD of up to 20% is often considered acceptable.^34^

The ANOVA assessed how well the regression model fits the data (Table S4). The *r* ^2^ value was 0.9982. As observed for the UAE, a regression model did not work well for predictions. There was a big difference between the lack of fit and pure error. This showed that the data did not fit the regression model as intended. Based on the results, we chose the extraction conditions from Experiment 3.

### Chemical Characterization of Tomato Leaves: Metabolite Annotation

Our optimized extraction protocols, coupled with dual-platform UHPLC-q-TOF-MS/MS and GC–MS analyses, proved highly effective in profiling the chemicals in both *S. pimpinellifolium* and *S. lycopersicum* (cultivar BRS Iracema). This approach enabled the robust detection and annotation of metabolites that are essential for understanding the impact of domestication and developmental stage on the chemistry of tomatoes. Tables S5 and S6 (see Supporting Information) provide detailed lists of the nonvolatile compounds identified by UHPLC-q-TOF-MS/MS and the primary and volatile compounds identified by GC–MS. These tables demonstrate the effectiveness of our methodology in capturing diverse chemical classes. The obtained chromatograms are illustrated in Figures S4–S6.

Specifically, UHPLC-q-TOF-MS/MS enabled the identification of key nonvolatile compounds, including various glycosylated alkaloids (e.g., alpha-tomatine, beta1-tomatine, esculeoside A, and *O*-glucosyl-tomatidine), phenylpropanoids (e.g., 3-*O*-feruloylquinic acid, caffeoylquinic acid, quercetin-3-*O*-rutinoside, and kaempferol-3-*O*-rutinoside), as well as gibberellins (e.g., Gibberellin A13). Following derivatization, GC–MS analysis successfully characterized numerous primary metabolites, including sugars (e.g., arabinose, galacturonic acid, and glucuronic acid), organic acids (e.g., malic acid,and glutaconic acid), and fatty acids (e.g., linoleic acid and stearic acid). Furthermore, the optimized HS-GC-MS approach effectively detected volatile compounds such as hexanal, alpha-pinene, hexanal, caryophyllene, and limonene, that contribute to distinct aroma profiles.

The robust detection of diverse metabolites ranging from highly polar primary metabolites to complex secondary compounds and volatiles underscores the effectiveness of our dual-platform analytical strategy in capturing the intricate complexity of the tomato metabolome. We used the identified compounds to characterize the differences and similarities among tomato species and their developmental stages.

### Insights into Chemical Differences Across Tomato Species

This unsupervised approach showed that tomato plants have more differences in chemical composition during the seedling stage than at other stages (Figure 1A–D). Seedling establishment is a fundamental stage in a plant’s life,^35^ involving intense and complex metabolic activity.^35,36^

**Figure 1:**
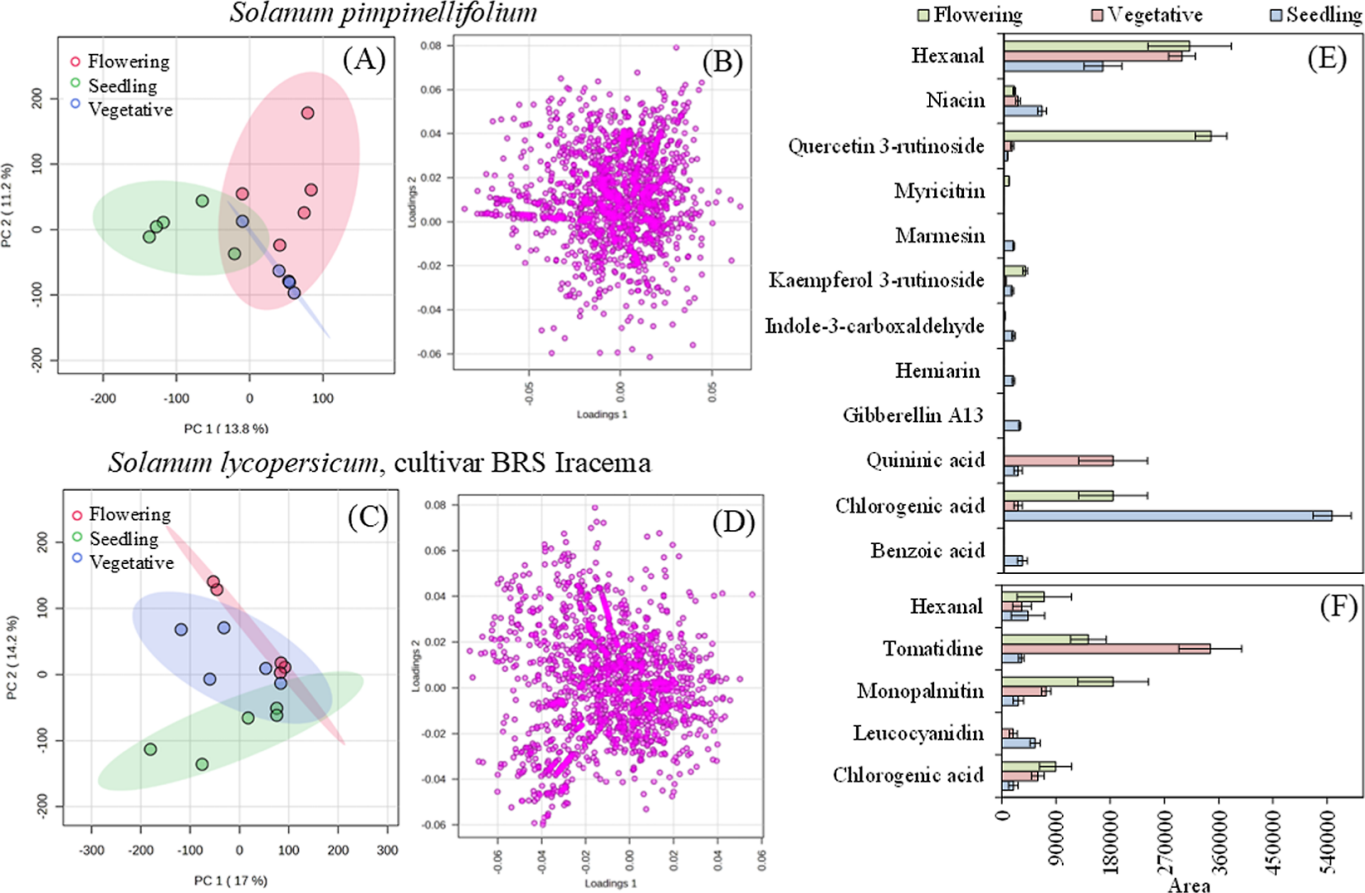
Plots of scores (A) and loadings (B) for the first and second components of the *Solanum pimpinellifolium* and *Solanum lycopersicum* (cultivar BRS Iracema) samples [(C) scores and (D) loadings] in the seedling, vegetative growth, and flowering stages. Compounds whose variation in area during the development stages was determinant for the grouping in the PCA analysis for the *Solanum pimpinellifolium* (E) and *Solanum lycopersicum* (cultivar BRS Iracema) (F) species.

We have selected specific molecules from Figure 1E,F to examine the metabolites that produce more variation in seedling stage species. One important nonvolatile compound, gibberellin A13 (GA13), was detected exclusively in seedlings of the wild species. Figure 2A shows the GA13 chromatogram and base peak mass spectrum. Other minor related compounds are present at low levels and do not interfere with the accurate identification of GA13.

**Figure 2:**
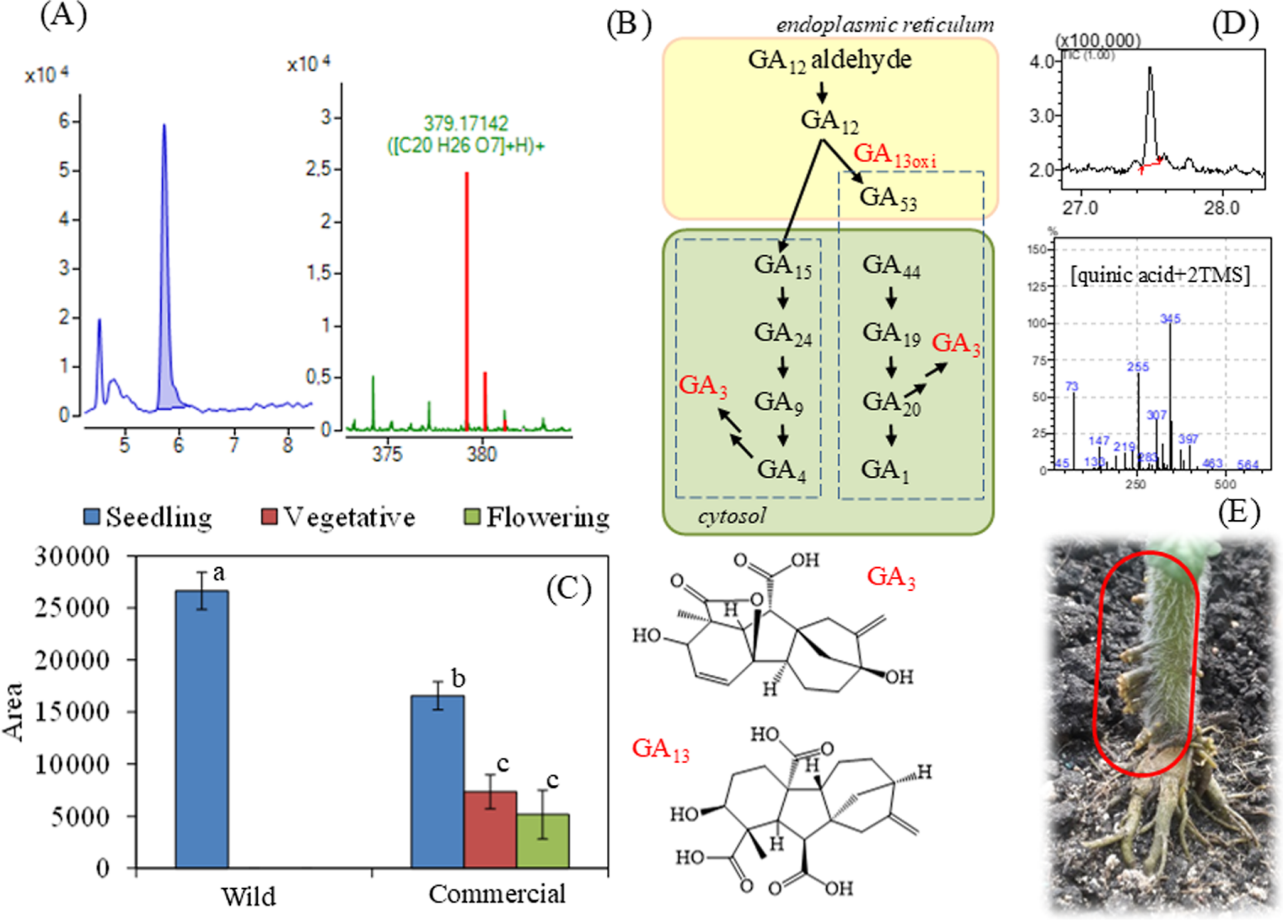
Chromatogram and mass spectrum highlighting the base peak mass of gibberellin A13 (A). Summary of gibberellin biosynthesis (B). Variation of gibberellin A13 area as a function of species and developmental stage (C). Different letters at the top of the bars indicate the statistical difference (*p* ≤ 0.05) between the means by Tukey’s test. Chromatogram and mass spectrum for chlorogenic acid (D). Representation of adventitious roots on tomato stem (E).

GA13 is a nonbioactive precursor that plays a crucial role in the biosynthesis of active 13-hydroxylated gibberellins (see Figure 2B for the biosynthesis pathway). GA13 begins in plastids with the cyclization of geranylgeranyl diphosphate (GGDP) into *ent*-kaurene. Oxidation steps, catalyzed by *ent*-kaurene oxidases and ent-kaurenoic acid oxidases, follow this initial step.^37^ Then, GA13 forms in the cytoplasm through a series of hydroxylation and oxidation reactions involving various dioxygenases.

Gibberellins are essential for various plant developmental processes in plants, including seed germination, stem elongation, and leaf expansion.^38,39^ The consistent level of GA13 across all growth stages in the commercial species, in contrast to its presence only in the wild seedling, suggests an alteration in gibberellin metabolism driven by domestication. Plant breeders often manipulate gibberellin pathways through genetic changes or exogenous applications to enhance desirable agronomic traits, such as faster germination, larger leaves for improved photosynthesis, and increased crop yields.^40^

Figure S8 shows hexanal, a compound present in both species but more abundant in the leaves of the wild species. This volatile metabolite is a product of the lipoxygenase pathway, an enzymatic process that breaks down unsaturated fatty acids. This pathway is found in all parts of the plant, including leaves and fruits.^41,42^ Hexanal is often associated with the perception of a “green” and “grassy” aroma in leaves.^43^ Fontes-Puebla et al. (2021)^44^ observed that selecting for desirable fruit characteristics during domestication often resulted in reduced lipoxygenase pathway activity when analyzing the profiles of these phytohormones and metabolites in plants representing evolutionary transitions from the wild corn ancestor to improved corn species. This genetic change can affect the production of volatiles, including hexanal, in both leaves and fruits.

Another major nonvolatile compound, chlorogenic acid, significantly affected species grouping, especially in the wild tomato. See Figure 1E for comparation of relative quantification (difference in area) and its chromatogram, and mass spectrum in Figure 2D. Yang et al. (2021)^45^ observed decreased levels of chlorogenic acid in cultivated diploid potato varieties compared to wild varieties. The authors suggest that this decrease may be the result of human selection because high concentrations of this molecule can negatively impact flavor, appearance, and other desirable commercial characteristics.

Additionally, this compound enhances stress resistance and root development.^46^ Mechanistically, chlorogenic acid and other phenolic compounds stimulate auxin activity, promoting root growth and lignin biosynthesis.^46,47^ Furthermore, chlorogenic acid is a precursor in the phenylpropanoid pathway that contributes to monolignols synthesis. Monolignols are the building blocks of lignin, which provides structural support to plant cell walls.^48^ Although commercial species often have more adventitious roots (Figure 2E), which is a critical trait for survival under stresses such as waterlogging^49^ and may be favored during breeding, this complex developmental process involves multiple molecular interactions beyond just chlorogenic acid.^50^

In the phenylpropanoid biosynthetic pathway, glycosylated forms of kaempferol and quercetin, such as rutin, influence how wild plants cluster in the PCA analysis (Figure 1E). The pathway begins with phenylalanine, which is deaminated to cinnamic acid by phenylalanine ammonia-lyase (PAL). Subsequent hydroxylation and methylation steps lead to various phenylpropanoid intermediates that ultimately form the backbone for flavonoids such as kaempferol and quercetin.^51^ These nonvolatile molecules act as potent antioxidants that protect against UV radiation and aid in plant defense against pathogens.^52^ The degree of glycosylation determines how cells transport these molecules.^53^ Although domestication can reduce their production in commercial plants, crossbreeding with wild species can reactivate flavonol biosynthesis.^54^

The tomato study revealed that the wild species exhibited different levels of glycosylated flavonols during its growth. In contrast, the commercial species showed changes in cyanidin production, displaying low levels of both cyanidin and its precursor, leukocyanidin (see Figure 1F). Inhibition of flavonol synthase, as demonstrated by Bovy et al. (2007),^52^ redirects metabolism from flavonols to anthocyanins, affecting fruit and flower color. This explains why the commercial species had more colorful flowers than the wild species (Figure 3). Specifically, flavonol synthase and dihydroflavonol 4-reductase (DFR) compete for dihydroflavonols. When flavonol synthase activity is inhibited, dihydroflavonols are diverted toward DFR, leading to anthocyanin biosynthesis (Figure 3).^55^

**Figure 3:**
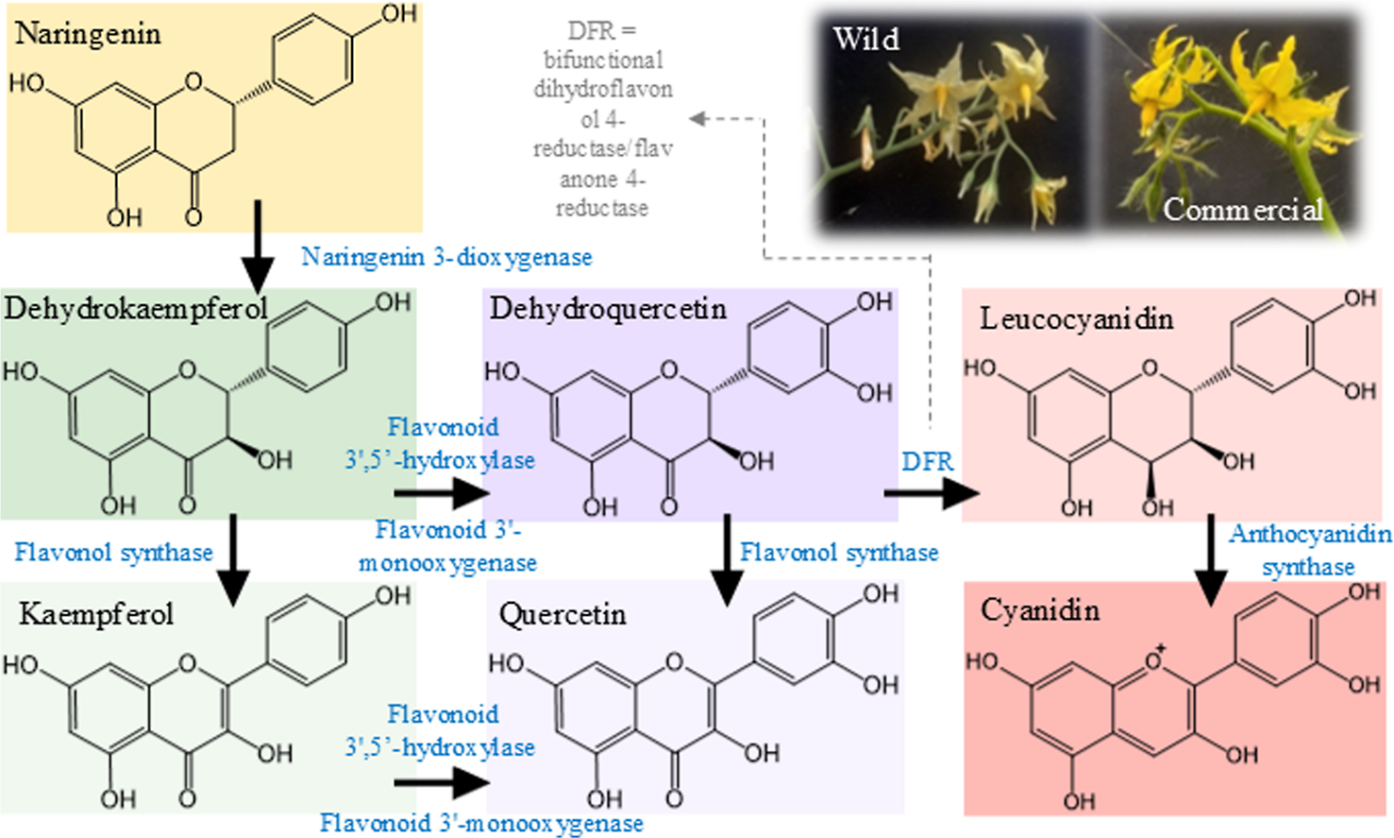
Diagram showing the formation of kaempferol and quercetin molecules from the naringenin molecule (phenylpropanoid pathway).

We have found that commercial plants have higher anthocyanin levels during their early development. During the early stages of plant growth, plants are under intense biochemical stress. This stress is associated with the development of roots and leaves. This may represent a strategy that maximizes the accumulation of polyphenols with high antioxidant activity, providing protection against potential abiotic and biotic stresses.^56^ This phenomenon has also been observed in sweet potato plants.^57^

Anthocyanins help in stems and leaves, and they also fight oxidation. They can make fruits last longer.^18^ However, many tomato types, including *S. lycopersicum* (commercial species), do not produce anthocyanins in their fruit. This observation has driven research efforts to develop species with enhanced fruit anthocyanin bioavailability, given their recognized antisclerotic, antitumor, and antidiabetic properties.^58^ Anthocyanin accumulation in tomato leaves is not only a result of domestication; it is also found in wild species.^52^ This shows that coordinated gene control plays a vital role in forming secondary metabolites in plants.

Another notable metabolite was tomatidine, a steroidal alkaloid derived from cholesterol (Figure 4A). This metabolite was more abundant in the wild species. Bai et al. (2025)^59^ demonstrated that DNA demethylation is associated with decreased levels of this metabolite during tomato domestication, resulting in fruits that are less bitter and toxic. The biosynthesis of steroidal alkaloids from cholesterol usually requires several enzymatic steps, such as hydroxylation, oxidation, and glycosylation reactions. These reactions are mediated by cytochrome P_450_ monooxygenases and glycosyltransferases.^60^ Tomatidine and its glycosides, with branched sugar chains, protect tomatoes from pests and diseases by inhibiting parasites like *Phytophthora infestans*.^61^ Glycosylated tomatidine exhibits strong antifungal activity^61^ and is present in both commercial and wild tomatoes.

**Figure 4:**
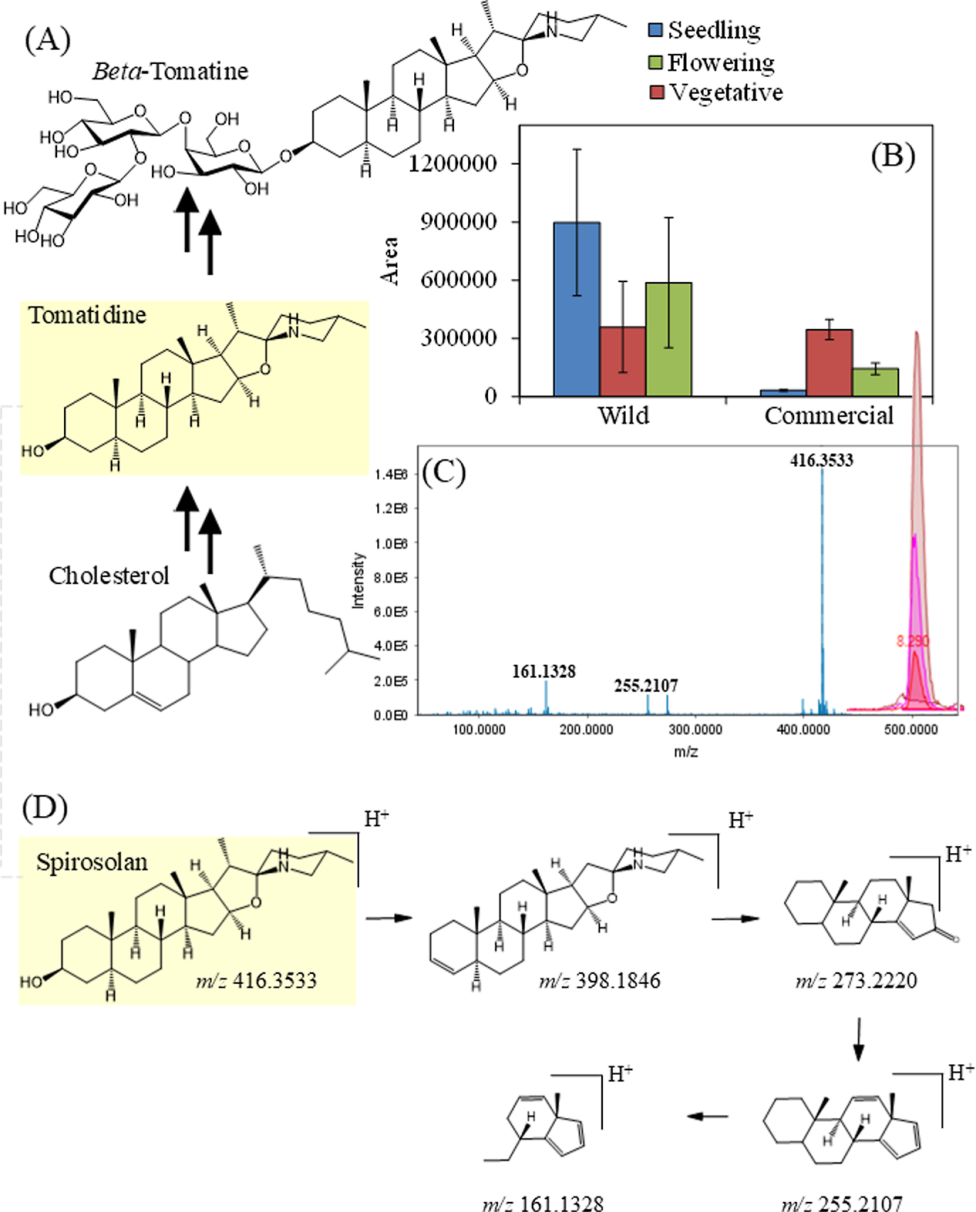
Summary of the biosynthesis of tomatidine from cholesterol (A). Modulation in the tomatidine pathway as a function of developmental stage and specie studied. Different letters at the top of the bars represent the statistical difference (*p* ≤ 0.05) between the means by Tukey’s test. (B). Peak and mass spectrum of tomatidine (C). Fragmentation levels of tomatidine (D).

Tomatidine levels changed during the growth stages of the commercial plants (Figure 4B). This change affected the grouping of samples from this species in the PCA analysis. The mass spectrum of tomatidine is shown in Figure 4C. The precursor ion [M + H]^+^ was assigned a *m*/*z* value of 416.3533. Fragmentation analysis revealed ions with *m*/*z* values of 398.1846, 273.2220, 255.2107, and 161.1328. The ion at 398.1846 represents water loss, labeled as [M – H_2_O]^+^. These ions come from the fragmentation of the spirosolane nucleus. This nucleus is the major structure of this class of alkaloids that has a secondary nitrogen. This fragmentation pattern shows a series of unsaturated hydrocarbons,^62^ as seen in Figure 4D.

### Chemical Profiling of Tomato Leaves Across Developmental Stages

This section compares the metabolic profiles of the evaluated species at three developmental stages. The metabolite analysis of both species at the seedling stage showed a clear separation in the PCA graph (Figure 5A). Next, we used a volcano plot. We found metabolites that showed significant differences between the two species (see Figure 5B). Figure 5C shows key metabolites that vary between the two species. Figure 5C shows a clear difference in alpha- and beta-tomatine levels between the two species at the seedling stage. Beta-tomatine is an intermediate in the biosynthesis of alpha-tomatine (Figure 6) and therefore has less biological activity compared to alpha-tomatine. However, it still contributes to abiotic stress resistance.^61^

**Figure 5:**
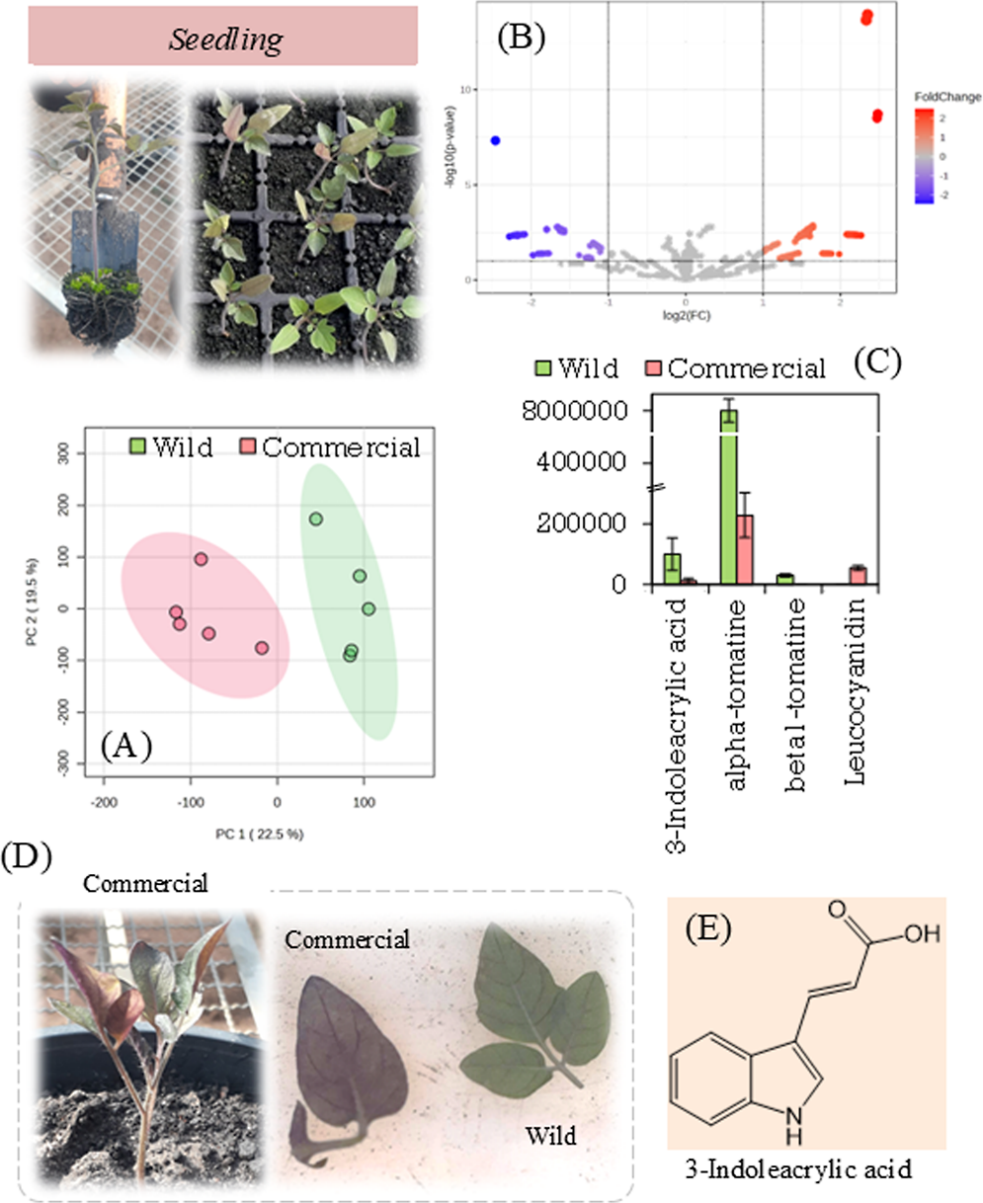
Photograph of plants at the seedling stage. Graph of PCA (A) and volcano (B) obtained by comparing the metabolites produced for plants at the seedling stage. Molecules differentially expressed by species (C). Photograph showing the difference in color of tomato leaves (D). Chemical structure of indole-3-acrylic acid (E).

**Figure 6:**
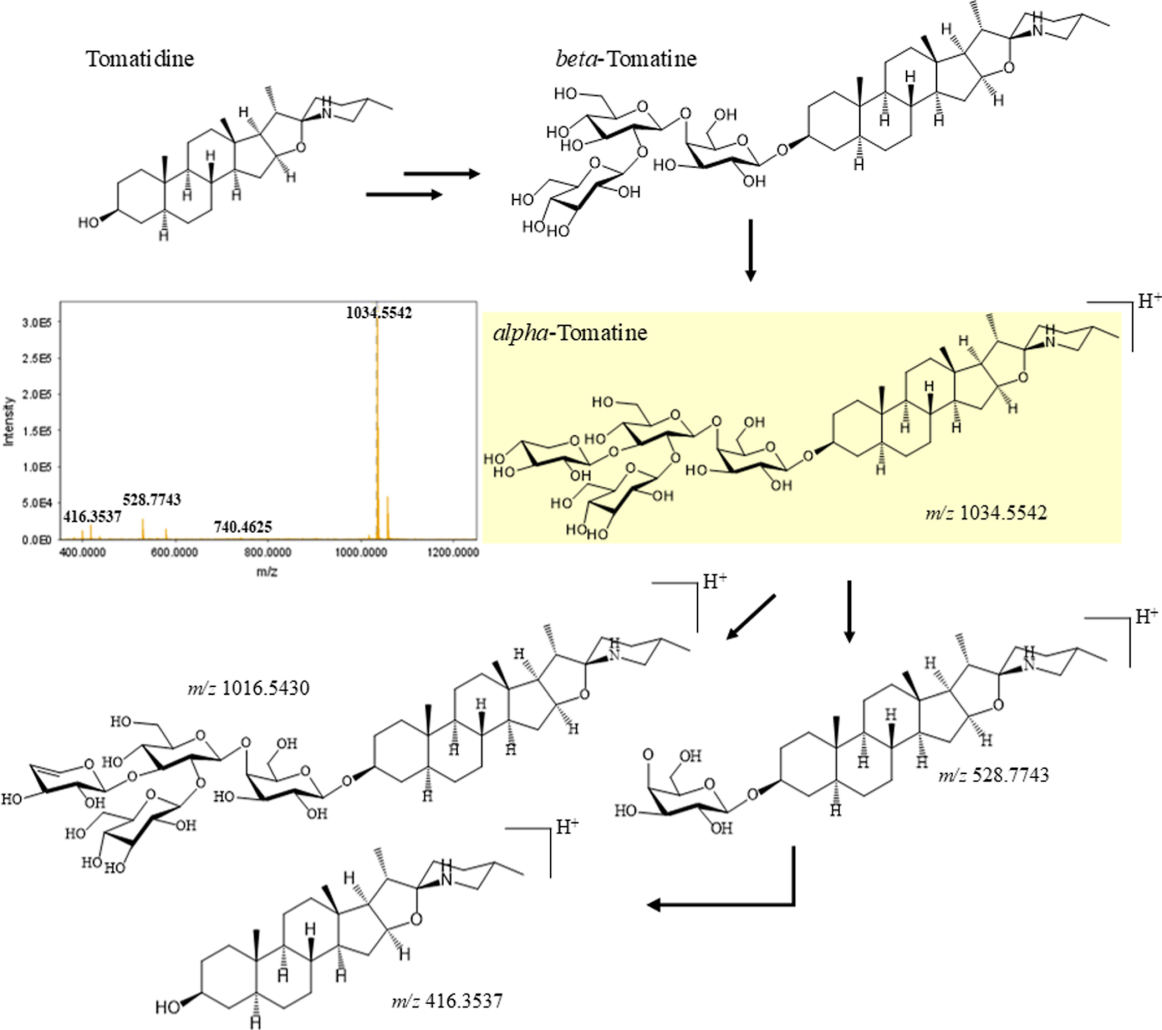
Steps of alpha-tomatine fragmentation.

Figure 6 illustrates some of the fragmentation steps observed for the alpha-tomatine molecule. The precursor ion [M + H]^+^ was assigned a *m*/*z* value of 1034.5542. The next fragmentation analysis produced an ion of *m*/*z* 1016.5430, which was attributed to water loss from the [M + H]^+^ ion. Fragmentation resulted in an ion of *m*/*z* 578.7749. This ion lost two d-glucose units and one xylose unit. It then lost all sugar units, resulting in an ion with an *m*/*z* of 416.3537. This last ion is the aglycone form, called tomatidine. These fragmentation pathways are shown in Figure 6.^63,64^

The commercial species has less alpha-tomatine than the wild species. It also has more tomatidine. This shows that tomato domestication has reduced glycoalkaloids, especially alpha-tomatine. This reduction probably occurs due to the focus on improving fruit flavor to appeal to consumers. Alpha-tomatine tastes bitter.^65^ This finding suggests that wild tomatoes contain more glycoalkaloids. Alpha-tomatine, which is abundant in green tissues and immature fruits, defends plants against pests and diseases.^66^ It and its aglycone are found in roots and modulate the rhizosphere microbiota, favoring beneficial bacteria and suppressing harmful ones in vitro.^67^

Figure 5C also clearly shows the detection of leukocyanidin. This isoflavone helped to identify samples from the commercial species at different growth stages. We found that this isoflavone and its anthocyanin derivatives appeared only in the commercial species when we examined each developmental stage separately. This finding may be related to the purple color seen in the leaves of the commercial species. In contrast, wild plants did not show this coloration (Figure 5D).

Figure 5E shows the chemical structure of 3-indoleacrylic acid. This molecule is more abundant in the wild species when it is in the vegetative stage. It may play a role in auxin metabolism. Consequently, 3-indoleacrylic acid may indirectly influence stem growth and plant morphological changes.^68^

Figure 7 shows plant growth in the vegetative stage. It includes the PCA plot (Figure 7A), a volcano plot (Figure 7B), and several differentially expressed metabolites (Figure 7C). During the vegetative growth phase, both species grew well. They developed a strong main stem and a large root system. There was also an increase in the number of leaves and branches. The vegetative stage requires many more nutrients than other stages.^69^ A fundamental molecule associated with energy gain in plants is glyceric acid. This is a important product of photosynthesis.^70^

**Figure 7:**
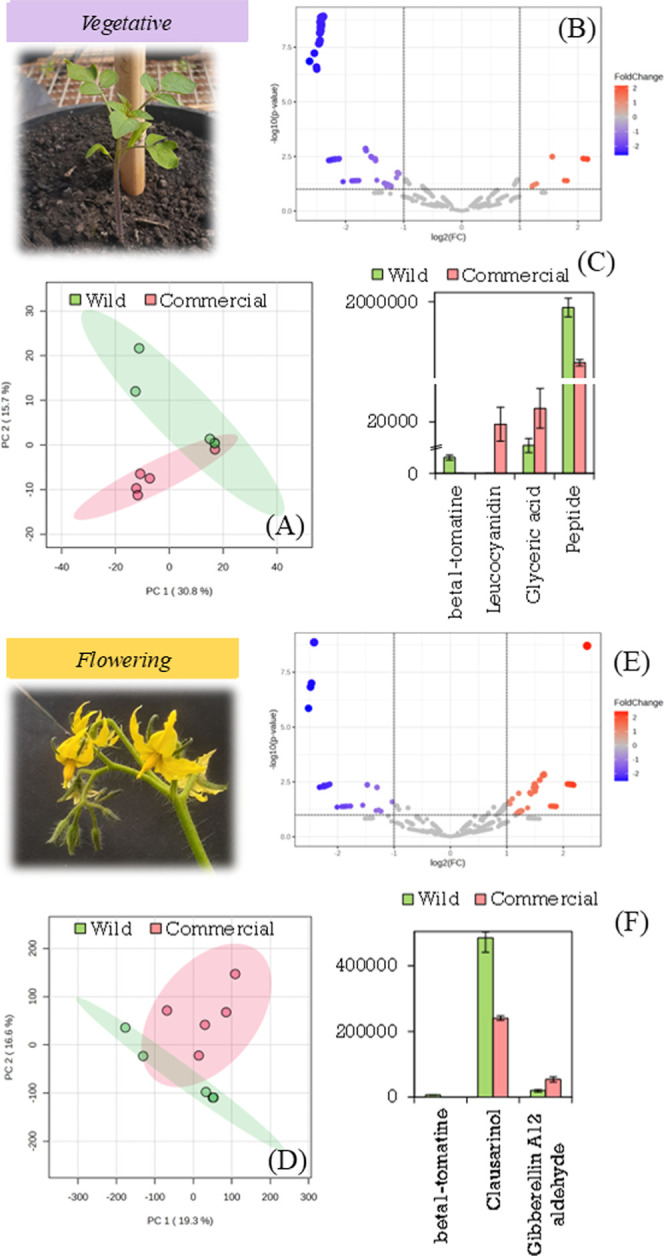
Photograph of plants at vegetative growth stage. Graph of PCA (A) and volcano (B) obtained by comparing the metabolites produced for plants at the seedling stage. Molecules differentially expressed by species (C). Photograph of plants at flowering stage. Graph of PCA (D) and volcano (E) obtained by comparing the metabolites produced for plants at the seedling stage. Molecules differentially expressed by species (F).

Glyceric acid and other organic acids are important in many plant metabolic pathways. Organic acid metabolism is essential for cells. It aids in energy production and forms the precursors needed for amino acids.^71^ Glyceric acid levels in Figure 7C were higher in the commercial species. Leaf size generally increases during plant domestication to increase photosynthetic capacity. The increase in leaf area may allow more carbon molecules to accumulate, such as organic acids.^72^

Another important finding is that the wild species has more of a specific peptide. These small peptides function as signaling molecules that influence plant development, including root growth.^73^ The specific peptide in our data remains incompletely characterized. However, preliminary analysis suggests that it is a peptide rich in tyrosine residues, with significantly higher levels observed in the wild species (see Figure S8). This observed difference in peptide abundance between the wild and commercial species may be due attributed to domestication processes.^74^

Figure 7 shows a photograph of the flowers of the commercial species. At this stage, the PCA analysis did not show a clear separation of the species (Figure 7D). During this stage, as in the previous one, plants send carbon to sink organs. These organs include roots, flowers, fruits, buds, and young leaves.^75^ The PCA analysis did not show clear groupings. A volcano plot (Figure 7E) showed which metabolites differed between the two species.

At this point, there was a clear difference in clausarinol levels between species. Clausarinol is a linear pyranocoumarin found in citrus fruits and apples (Figure 7F).^76^ Most plants do not accumulate pyranocoumarins. Coumarins, when active, help plants fight off phytopathogens. Roots are the main producers of these molecules. They can alter soil microbial communities and help with iron uptake.^77^

Another molecule that showed significant differential expression between the species was gibberellin aldehyde A12. This compound is an intermediate in the main biosynthetic pathway of gibberellins. In flowering plants, the proper formation and growth of inflorescences is crucial. These reproductive organs help ensure the survival of the species. This process is controlled by a mixture of phytohormones. These include gibberellins, jasmonates, auxins, and cytokinins. These hormones must be present and balanced for flowers to develop properly.^78^ Gibberellins are essential for the growth of almost every plant organ. They help cells divide and grow longer. They are the key to development. This includes seed germination and the transition from vegetative to reproductive growth.^79^

## Conclusion

Using an optimized, integrated chemical approach, this study thoroughly characterized the chemical profiles of two tomato species, wild (*S. pimpinellifolium*) and commercial (*S. lycopersicum*, cultivar BRS Iracema), across three critical developmental stages. Our results clearly demonstrate that domestication altered the tomato metabolome, resulting in significant chemical differences between the wild ancestor and modern species. Wild tomato leaves consistently exhibited higher concentrations of defense-related compounds, such as the nonvolatile glycoalkaloids alpha-tomatine and tomatidine, especially during the seedling stage. We also detected higher levels of volatile metabolites, such as hexanal, in the wild species. In contrast, the commercial species displayed improved anthocyanin and primary metabolite biosynthesis, such as glyceric acid production, indicating adaptive changes geared toward growth and fruit quality. While developmental stage influenced metabolite profiles, domestication was the most significant factor in shaping the overall chemical landscape. Our results shed light on the biochemical implications of domestication and indicate areas for further exploration in agricultural improvement.

## Supplementary Material



## Notes

Planning matrix for extraction of fixed compounds by UAE and dependent responses (Table S1). Plots of the effects of the variables studied on the UAE. Chromatogram of the total extracted compounds for the samples (Figure S1). Variance analysis for linear model adjustment (95% confidence level) for UAE (Table S2). Display of chromatograms obtained after derivatization and analysis by GC–MS. (Figure S2). Planning matrix for headspace extraction and dependent responses (Table S3). Representation of the effects of the studied variables on the headspace extraction (Figure S3). Variance analysis for linear model adjustment (95% confidence level) for headspace (Table S4). Chromatogram of the total extracted compounds for the nonvolatile (Figure S4), derivatized (Figure S5) and volatile (Figure S6) compounds from the leaves of *S. pimpinellifolium* and the BRS Iracema cultivar of *S. lycopersicum* at three developmental stages: seedling, vegetative, and flowering. Compounds detected in tomato leaves by UHPLC-q-TOF-MS/MS (Table S5). Compounds detected in tomato leaves by GC–MS (Table S6). Variation of hexanal area as a function of species and developmental stage (Figure S7). Mass spectrum obtained for the peptide with emphasis on tyrosine fractionation (Figure S8) (PDF)
