# Supplementary material for: Chemical Profiling of Wild and Commercial Tomato Leaves: Protocol Optimization, Insights into Differences in Chemical Profiles between Species and Developmental Stages
Source: ACS Omega. 2025 Sep 18;10(38):43632–44. doi: 10.1021/acsomega.5c03637 (PMC12489728; doi:10.1021/acsomega.5c03637)
Supplement: Supplementary file 1 [file ao5c03637_si_001.pdf]

Supporting Information

Chemical Profiling of Wild and Commercial Tomato Leaves: Protocol Optimization, Insights into Differences in Chemical Profiles Between Species and Developmental Stages

Maria Clara Santana Aguiar, Moacir Rossi Forim*

[a]*Department of Chemistry, Universidade Federal de São Carlos, Rod. Washington Luiz Km 235 s/n, Postal Code 13565-905, São Carlos - SP, Brazil.*

*Corresponding author

Moacir Rossi Forim, Tel: +55 16 3351.8061; Fax: +55 16 3351.8350

e-mail: *mrforim@ufscar.br*

Table S1. Planning matrix for extraction of fixed compounds by UAE and dependent responses. Individual and global desirability values obtained by evaluating the extractor mixture and time in the UAE.

Essay	Extract mixture[a]	Time[b]	*Molecular features*		Total area[c]			Extract mixture	Time	D
			1[d]	2[d]	1[d]	2[d]	Sum (Σ)	d	d	
1	1 (70%)	1 (35)	3165	2354	2.19×10^{8}	3.58×10^{9}	3.79×10^{9}	0.75	0.13	0.44
2	-1 (30%)	1(35)	2632	2391	3.15×10^{9}	3.11×10^{9}	6.26×10^{9}	0.00	0.52	0.26
3	1 (70%)	-1 (5)	2906	2682	2.54×10^{8}	2.74×10^{9}	2.99×10^{9}	0.38	0.00	0.19
4	-1 (30%)	-1 (5)	3017	2181	3.23×10^{9}	3.30×10^{9}	6.52×10^{9}	0.54	0.57	0.55
5	0 (50%)	0 (20)	3288	2329	4.41×10^{9}	2.99×10^{9}	7.40×10^{9}	0.92	0.71	0.81
6	0 (50%)	0 (20)	3285	2235	4.57×10^{9}	3.20×10^{9}	7.77×10^{9}	0.91	0.76	0.84
7	0 (50%)	0 (20)	3346	2449	5.36×10^{9}	3.88×10^{9}	9.24×10^{9}	1.00	1.00	1.00
8	0 (50%)	0 (20)	3168	2146	4.38×10^{9}	4.08×10^{9}	8.46×10^{9}	0.75	0.87	0.81

a = ethanol solution; b = time in minutes; c = sum of the areas of the compounds obtained in the first and second extraction cycles; d = the numbers 1 and 2 refer to the first and second extraction cycles; d = individual desirability; D = global desirability.

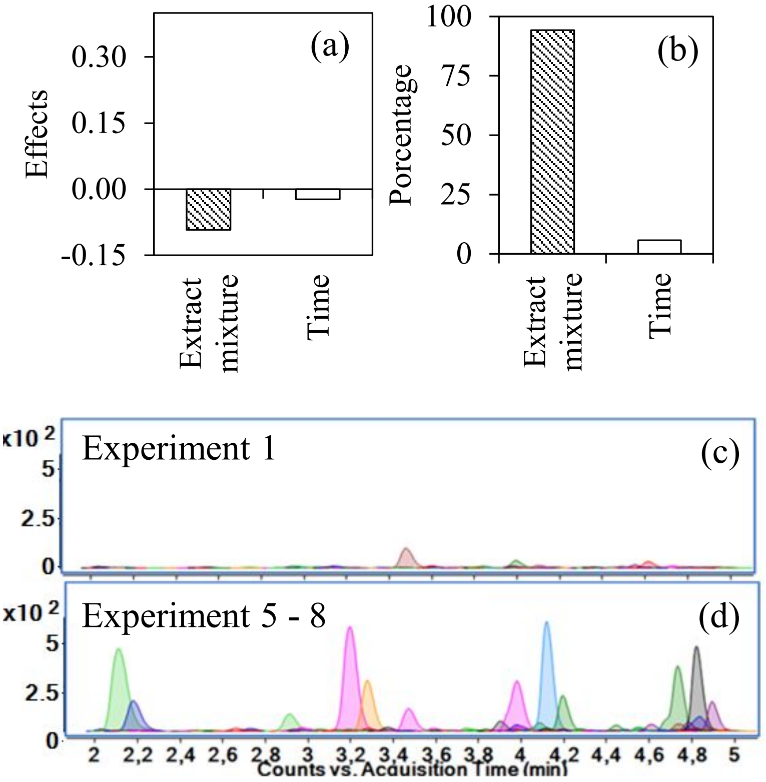

Figure S1. Plots of the effects of the variables studied on the UAE. The variables evaluated were the extractor mixture, the extraction time and the interaction between the two (a-b). Chromatogram of the total extracted compounds for the samples obtained in experiment 1 and in the central point experiments (5 to 8) (c-d).

Table S2. Variance analysis for linear model adjustment (95% confidence level) for UAE.

Source of variation	Sum of squares	Degrees of freedom	Mean squares	F	F_{tab}
Regression	0.0809	3	0.0270	0.2015	6.59
Residual	0.5354	4	0.1338		
Total	0.6163	7	0.0880		
Pure error	0.0244	4	0.081	62.79	10.13
Lack of fit	0.5110	1	0.510		
r^2	0.9604				

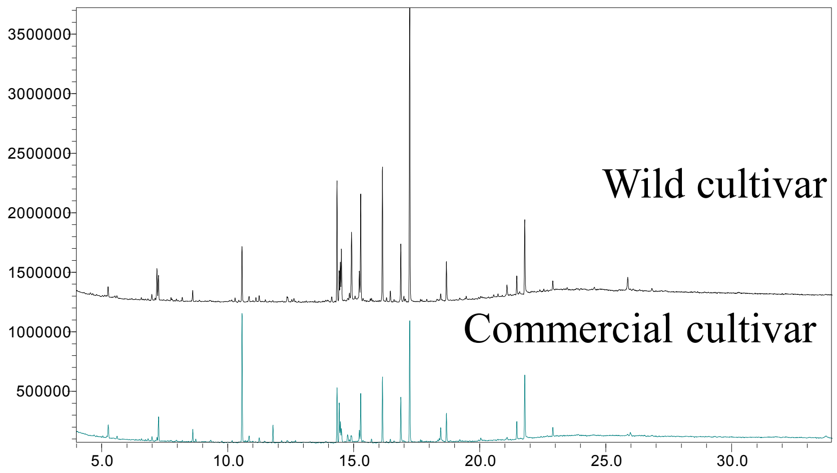

Figure S2. Display of chromatograms obtained after derivatization and analysis by GC-MS.

Table S3. Planning matrix for headspace extraction and dependent responses. Individual and global desirability values obtained when evaluating ionic strength and time in headspace extraction.

Essay	Ionic strength[a]	Time[b]	Number of compounds	Total area	Compounds	Área	Dg
					di	di	
1	1	1	25	4.22×10^{6}	0.80	0.33	0.57
2	-1	1	20	2.15×10^{6}	0.55	0.12	0.34
3	1	-1	29	1.09×10^{7}	1.00	1.00	1.00
4	-1	-1	9	9.02×10^{5}	0.00	0.00	0.00
5	0	0	11	1.86×10^{6}	0.10	0.10	0.10
6	0	0	13	1.40×10^{6}	0.20	0.05	0.12
7	0	0	10	2.06×10^{6}	0.05	0.11	0.08
8	0	0	13	1.47×10^{6}	0.20	0.06	0.13

a = NaCl solution; b = time in minutes; d = individual desirability; D = global desirability.

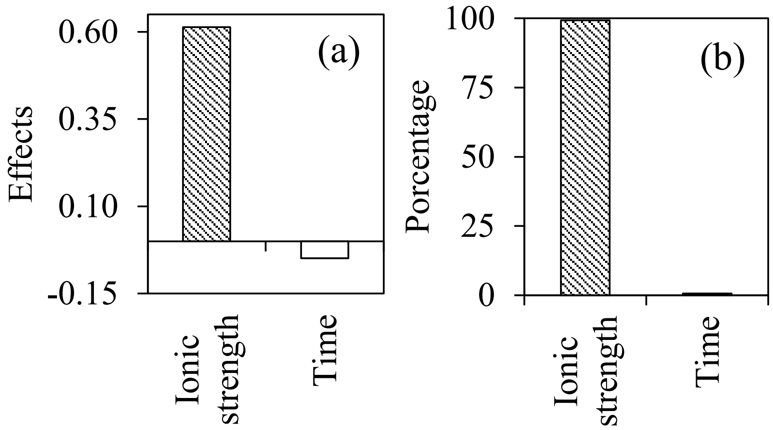

Figure S3. Representation of the effects of the studied variables on the headspace extraction. The variables ionic strength and extraction time were evaluated.

Table S4. Variance analysis for linear model adjustment (95% confidence level) for headspace.

Source of variation	Sum of squares	Degrees of freedom	Mean squares	F	F_{tab}
Regression	0.5284	3	0.1761	2.5955	6.59
Residual	0.2714	4	0.0679		
Total	0.7998	7	0.1143		
Pure error	0.0014	4	0.0005	566.84	10.13
Lack of fit	0.2799	1	0.2700		
r^2	0.9982				

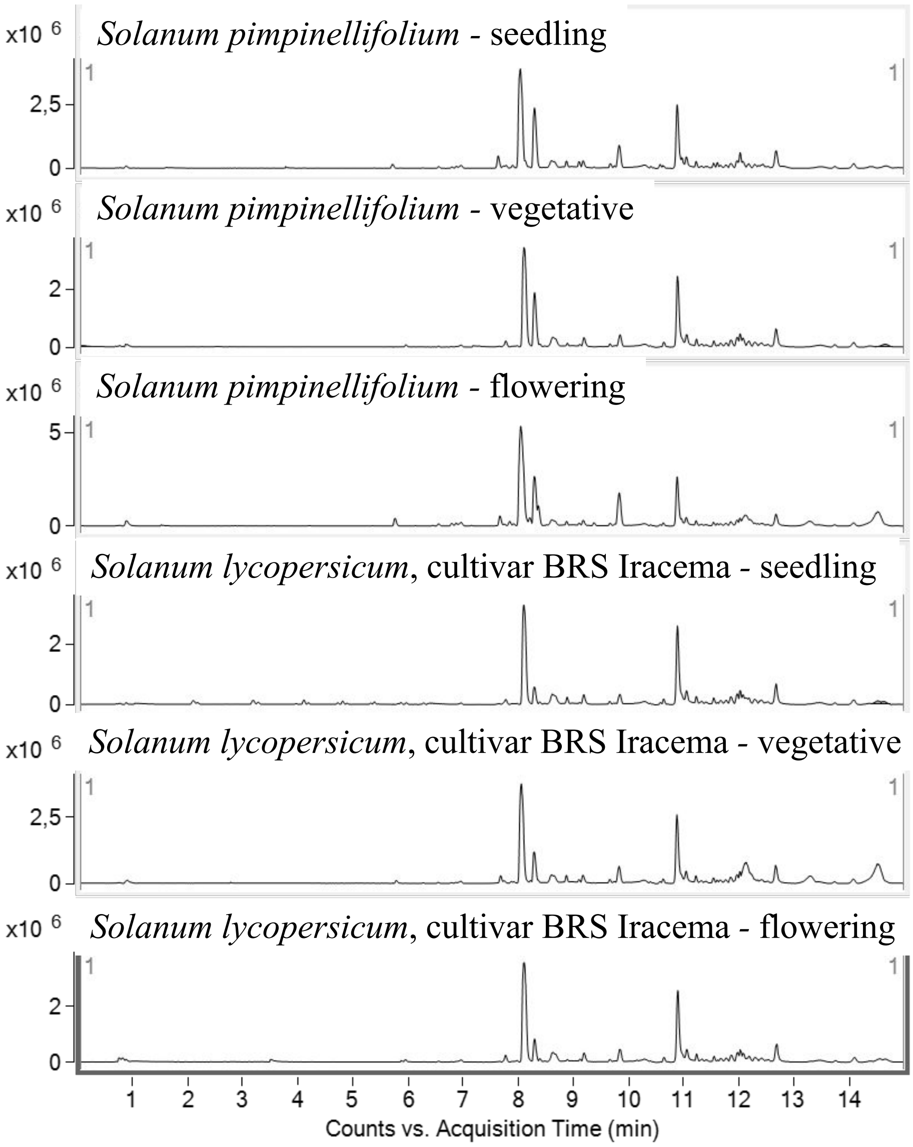

Figure S4. A comparison of the total ion chromatograms of non-volatile compounds from the leaves of *Solanum pimpinellifolium* and the BRS Iracema cultivar of *Solanum lycopersicum* at three developmental stages: seedling, vegetative, and flowering.

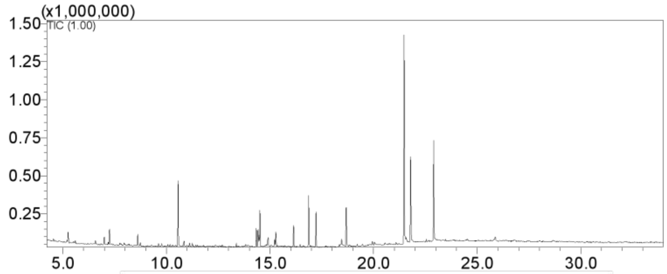

Solanum pimpinellifolium - vegetative

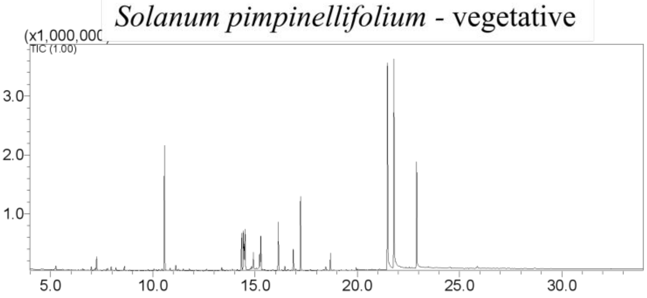

Solanum pimpinellifolium - flowering

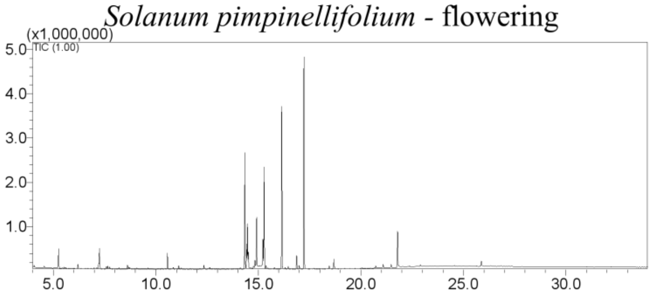

Solanum lycopersicum, cultivar BRS Iracema seedling

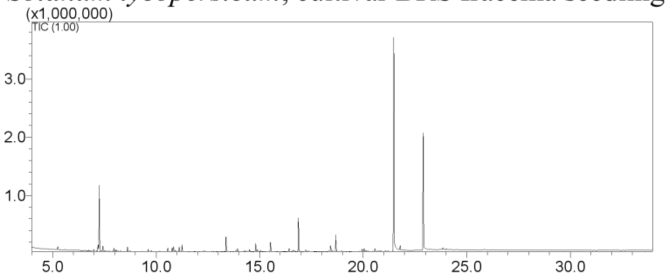

Solanum lycopersicum, cultivar BRS Iracema - vegetative

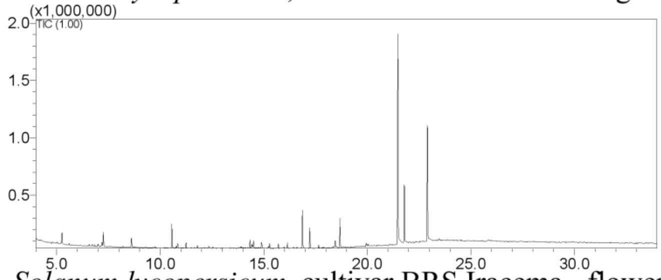

Solanum lycopersicum, cultivar BRS Iracema - flowering

Figure S5. A comparison of the total ion chromatograms of derivatized compounds from the leaves of *Solanum pimpinellifolium* and the BRS Iracema cultivar of *Solanum lycopersicum* at three developmental stages: seedling, vegetative, and flowering.

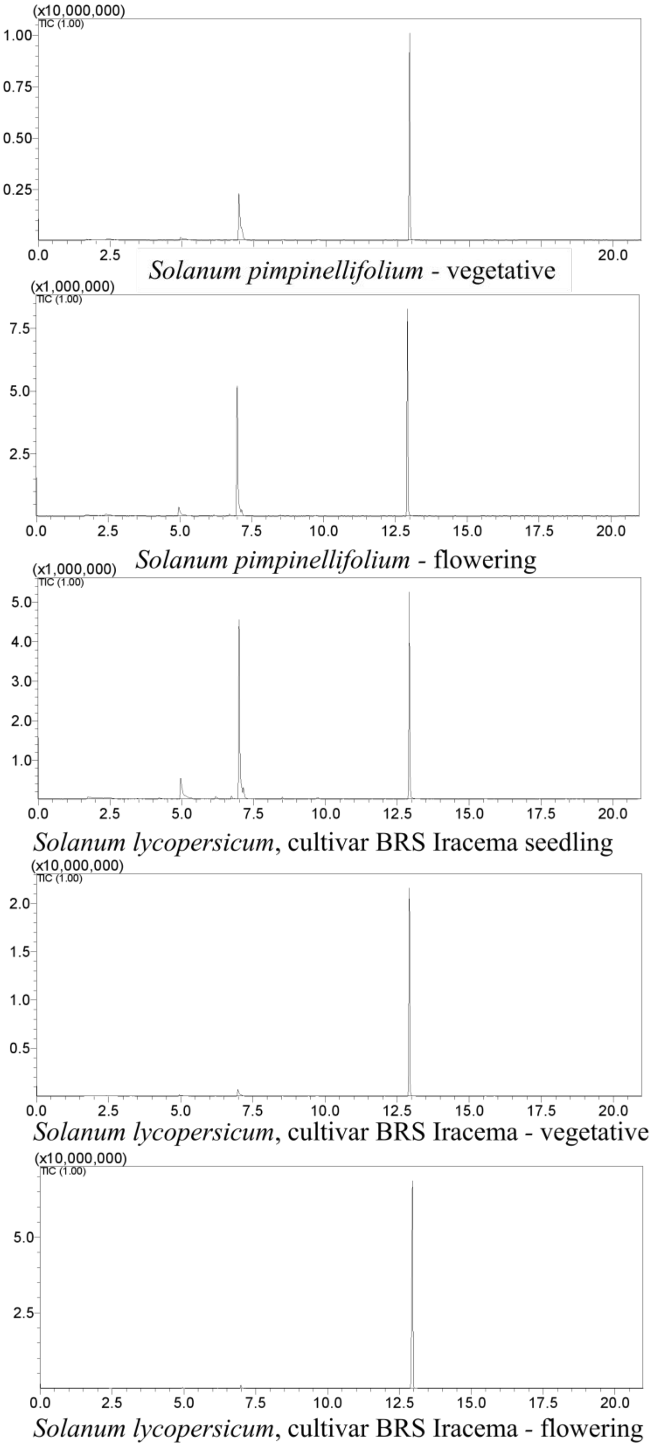

Figure S6. A comparison of the total ion chromatograms of volatile compounds from the leaves of *Solanum pimpinellifolium* and the BRS Iracema cultivar of *Solanum lycopersicum* at three developmental stages: seedling, vegetative, and flowering.

Table S5. Compounds detected in tomato leaves by UHPLC-q-TOF-MS/MS.

Putative ID	Main fragments	Precursor ions detected			Molecular formula	Error (ppm)
		$(M+H)^+$	$(M+Na)^+$	$(M+K)^+$		
3-*O*-Feruloylquinic acid	160.07	368.1109	386.1446	-	$C_{17}H_{20}O_9$	-0.53
7-Methoxycoumarin	121.06<br>118.03	177.0548	-	-	$C_{10}H_8O_3$	-0.99
alpha-Tomatine	1016.54<br>528.77<br>416.35	1034.5609	1056.5359	-	$C_{50}H_{83}NO_{21}$	0.52
*beta*1-Tomatine	416.35	902.5117	-	-	$C_{45}H_{75}NO_{17}$	1.24
Caffeoylputrescine	163.03<br>135.04	251.1389	-	-	$C_{13}H_{18}N_2O_3$	-0.75
Caffeoylquinic acid	163.03	354.0955	377.0848	-	$C_{16}H_{18}O_9$	-1.29
Coumarin	119.04<br>91.05	147.0445	-	-	$C_9H_6O_2$	-1.15
Cyanidine	241.04<br>213.05	287,0560	-	-	$C_{15}H_{11}O_6^+$	1.74
Esculeoside A	-	1270.6068	1292.5856	-	$C_{58}H_{95}NO_{29}$	0.50
Feruloylputrescine	248.96<br>177.05	265.1544	-	-	$C_{14}H_{20}N_2O_3$	0.97
Gamma-aminobutyric acid	86.06<br>69.03	104.0709	-	-	$C_4H_9NO_2$	2.88
Gibberellin A13	333.14<br>287.10	379.1450	-	-	$C_{20}H_{26}O_7$	1.67
Indole	100.12<br>90.04	118.0652	-	-	C_8H_7N	-1.34
Indole-3-carboxaldehyde	118.06<br>90.04	146.0602	-	-	C_9H_7NO	-1.23
Indoleacrylic acid	170,06<br>132,08	188,0709	-	-	$C_{11}H_9NO_2$	-1.59

Kaempferol-3-*O*-rutinoside	287.10	595.1632	-	-	$C_{27}H_{30}O_{15}$	3.54
Leucocyanidin	163.04<br>155.04	307.0813	-	-	$C_{15}H_{14}O_7$	0.32
L-Phenylalanine	149.05<br>131.04<br>120.07	166.0864	-	-	$C_9H_{11}NO_2$	-0.6
Methoxyisoflavone	-	342.0737	365.0635	-	$C_{18}H_{14}O_7$	0.77
O-glucosyl-tomatidine	255.21<br>161.13<br>147.11	578.4067	-	-	$C_{33}H_{55}NO_7$	1.57
p-Coumaroyl putrescine	218.12<br>119.04	235.1437	-	-	$C_{13}H_{18}N_2O_2$	1.18
Quercetin-3-*O*-rutinoside	303.04	611.1648	633.1389	-	$C_{27}H_{30}O_{16}$	-1.14
Solasodine glucoside	253.19<br>157.10	576.3898	-	-	$C_{33}H_{53}NO_7$	0.32
Tomatidine	398.18<br>273.22<br>255.21<br>161.13	416.3516	-	-	$C_{27}H_{45}NO_2$	2.17
Tripeptide	-	420.1870	-	-	$C_{19}H_{25}N_5O_6$	0.38
Tripeptide formed from L-histidyl, L-serine and L-valine residues	-	342.1759	-	-	$C_{14}H_{23}N_5O_5$	3.49
Umbelliferone	134.03<br>79.05	163.0390	-	-	$C_9H_6O_3$	-0.77
Xanthine	200.04<br>145.04	-	-	219.0265	$C_7H_8N_4O_2$	-1.36
Xanthyletin	213.05<br>133.10	229.0857	-	-	$C_{14}H_{12}O_3$	-0.92

Table S6. Compounds detected in tomato leaves by GC-MS.

Putative ID	Main fragments	Molecular formula
1-Penten-3-ol	57, 41, 43	$C_5H_{10}O$
2-Keto-isovaleric acid-oxime (2TMS)	231, 147, 100, 73	$C_{11}H_{25}NO_3Si_2$
3-Hydroxybutyric acid (2TMS)	233, 147, 73	$C_{10}H_{24}O_3Si_2$
3-Methylglutaconic acid (2TMS)	273, 183, 147, 73	$C_{12}H_{24}O_4Si_2$
alpha-pinene	121, 93, 77, 53	$C_{10}H_{16}$
Arabinose (4TMS)	217, 204, 191, 147, 73	$C_{17}H_{42}O_5Si_4$
Benzyl alcohol	108, 79, 51	C_7H_8O
Caffeic acid (TMS)	396, 381, 307, 249, 219, 191, 73	$C_{18}H_{32}O_4Si_3$
Caryophyllene	189, 133, 91, 79	$C_{15}H_{24}O$
Chlorogenic acid (6TMS)	345, 255, 73	$C_{16}H_{18}O_9$
D-Limonene	121, 94, 79, 67, 68	$C_{10}H_{16}$
Galacturonic acid (5TMS)	217, 147, 103, 73	$C_{21}H_{50}O_7Si_5$
Glucuronic acid (5TMS)	331, 305, 217, 204, 147, 73	$C_{21}H_{50}O_7Si_5$
Glutaconic acid (2TMS)	217,169, 147, 73	$C_{11}H_{22}O_4Si_2$
Hexanal	72, 57, 44	$C_6H_{12}O$
Linoleic acid (TMS)	337, 262, 129, 117, 95, 81, 73, 55	$C_{21}H_{40}O_2Si$
Malic acid (3TMS)	149, 148, 147, 115, 99, 75, 73	$C_{13}H_{30}O_5Si_3$
Phenylethyl Alcohol	122, 91, 77, 65	$C_8H_{10}O$
Stearic acid (TMS)	341, 297, 145, 129, 117, 73	$C_{21}H_{44}O_2Si$

Threitol (4TMS)	217, 205, 147, 103, 73	$C_{16}H_{42}O_4Si_4$
Threonic acid (4TMS)	319, 292, 205, 147,73	$C_{16}H_{40}O_5Si_4$
trans-3-Hexen-1-ol	100, 82, 67	$C_6H_{12}O$
trans-Hex-2-enal	97, 83, 69, 41	$C_6H_{10}O$

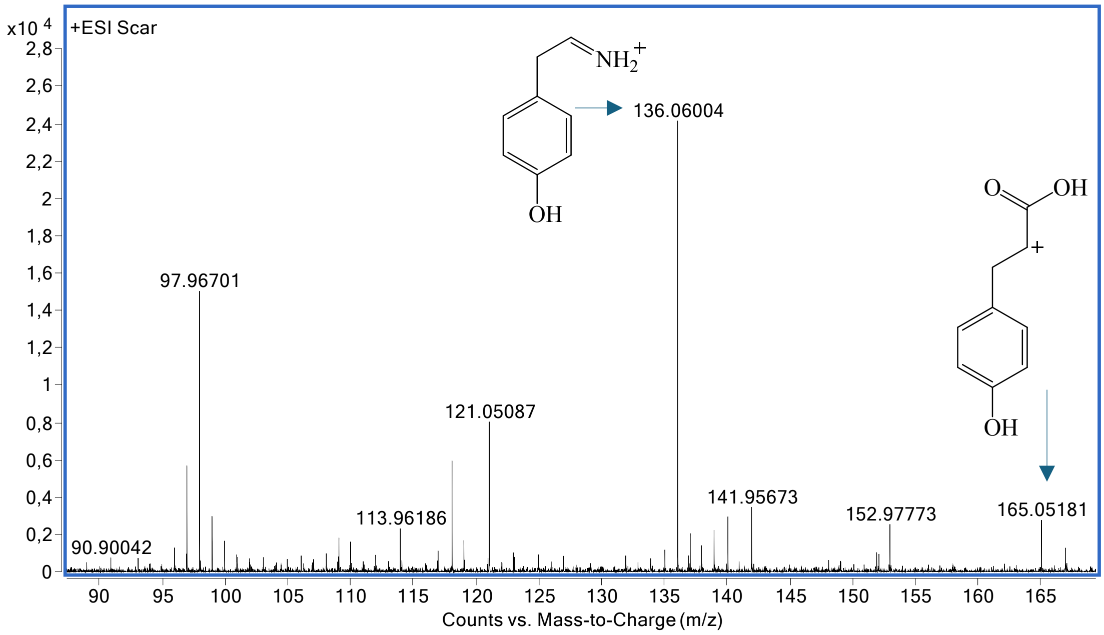

Figure S7. Mass spectrum obtained for the peptide with emphasis on tyrosine fractionation.
